# First-Principles Insights into Li Storage and Ion Diffusion in B‑, P‑, and S‑Doped C_2_N Anodes

**DOI:** 10.1021/acsomega.5c12977

**Published:** 2026-04-01

**Authors:** Fereshteh Ghorbani Shadpey, Maryam Soleimani, Mahdi Pourfath

**Affiliations:** † School of Electrical and Computer Engineering, College of Engineering, University of Tehran, Tehran 14395-515, Iran; ‡ Institute for Microelectronics/E360, 108871 TU Wien, A-1040 Vienna, Austria

## Abstract

Two-dimensional C_2_N monolayers are promising anode materials for lithium-ion batteries due to their high nitrogen content, intrinsic porosity, and tunable electronic properties. In this work, first-principles density functional theory (DFT) is used to systematically investigate B-, P-, and S-doped C_2_N monolayers at two dopant concentrations (2.78 and 5.56 at. %). Doping substantially modifies the electronic structure, closes the band gap, and enhances Li adsorption without compromising overall structural stability. B-doped C_2_N exhibits the highest thermodynamic favorability for incorporation (*E*_*f*_ = 1.36 eV), while S-doping introduces lattice flexibility and facilitates fast Li diffusion with a low energy barrier of ∼0.36 eV, markedly lower than that of pristine C_2_N. The average open-circuit voltage increases to 2.93 and 2.77 V for P- and B-doped systems, respectively, compared to pristine C_2_N (∼2.18 V), while S-doped C_2_N maintains a moderate voltage of 2.12 V with enhanced rate capability. High dopant concentrations further increase Li storage but introduce diffusion anisotropy, highlighting a trade-off between capacity and ion mobility. This study provides critical insights and design guidelines for developing high-capacity, fast-charging C_2_N-based anodes.

## Graphical abstract

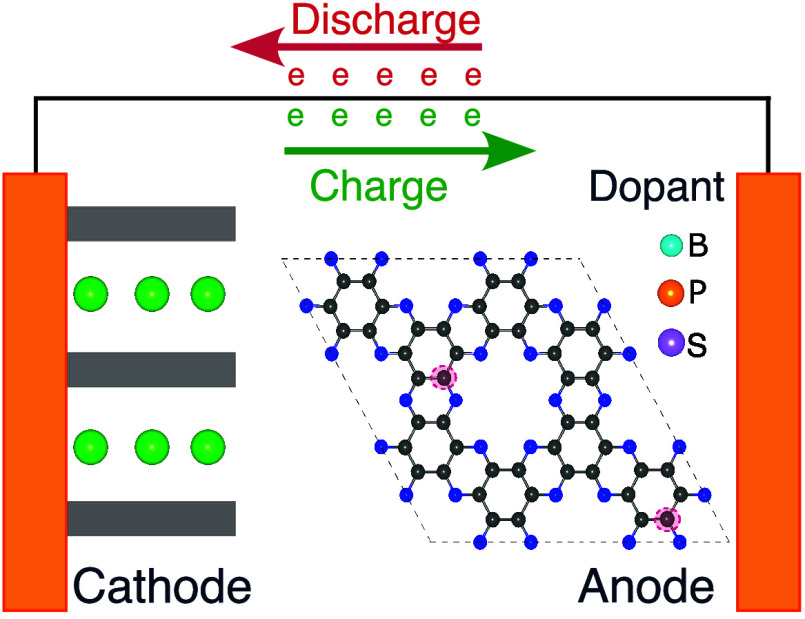

## Introduction

The rapid expansion of portable electronics, electric vehicles, and renewable energy systems has created strong demand for rechargeable batteries that combine high energy and power densities with long cycle life and reliable safety. Among the available electrochemical energy storage technologies, lithium-ion batteries dominate due to their high voltage, energy density, efficiency, and relatively long cycle life.^1,2^ However, to meet the performance requirements of modern applications, significant improvements in the electrochemical properties of electrode materials—particularly the anode—are needed.^3^

Graphite, the conventional anode material in commercial lithium-ion batteries, is limited by its relatively low theoretical capacity of 372 mAh g^–1^, slow lithium diffusion kinetics, and potential safety concerns at high charging rates. These limitations motivate the search for new anode materials with higher lithium storage capacity, faster rate performance, and stronger structural integrity during lithiation/delithiation processes.^4^

Two-dimensional (2D) materials have emerged as promising anode candidates due to their atomic thickness, large specific surface area, short ion diffusion pathways, and tunable electronic properties.^4,5^ Porous carbon nitrides are particularly attractive because of their high nitrogen content, chemical robustness, and structural versatility.^6,7^

The C_2_N monolayer, also known as nitrogenated holey graphene, represents a unique member of this family. It was first synthesized via a bottom-up wet-chemical route by Baek and co-workers, yielding a highly crystalline 2D carbon nitride framework with periodic nanopores.^7,8^ C_2_N consists of benzene rings bridged by pyrazine units, forming a porous lattice that facilitates lithium diffusion and adsorption. First-principles calculations suggest that C_2_N can achieve a high theoretical lithium storage capacity of up to ∼667 mAh g^–1^, surpassing graphite, while maintaining structural stability during lithiation.^8^ Favorable diffusion barriers and open-circuit voltages have been reported for Li and Na storage.^9–14^ Experimentally, C_2_N-based electrodes demonstrate high reversible capacities and stable cycling in lithium-ion batteries.^8^ Hybrid architectures, such as C_2_N confined on reduced graphene oxide (C_2_N/rGO), further enhance conductivity and long-term stability, highlighting the potential of C_2_N as a functional component in composite or hybrid anodes to optimize electrochemical performance.^15^ Metal-decorated C_2_N composites further demonstrate enhanced lithium storage and tunable performance.^16^ Despite these promising characteristics, pristine C_2_N possesses a relatively wide band gap (>1.5 eV), limiting its intrinsic electronic conductivity.^7,17^

Substitutional doping has proven to be an effective approach for tuning the electronic and chemical properties of 2D materials without compromising structural integrity.^18–20^ In graphene-derived systems, incorporation of nonmetal heteroatoms significantly modifies the density of states, enhances conductivity, and strengthens ion adsorption.^21–27^ For C_2_N, substitutional doping with boron (B), phosphorus (P), and sulfur (S) has been reported to influence lattice connectivity, induce charge redistribution, and enhance surface reactivity.^19,28^ Doped C_2_N structures exhibit stronger adsorption interactions and modified electronic properties compared to the pristine monolayer. First-principles studies show that B-, P-, and S-doped C_2_N possess higher methoxyphenol adsorption energies than pristine C_2_N, reflecting enhanced chemical reactivity and adsorption capacity.^19^ Additionally, another study indicates that B substitution in C_2_N is thermodynamically feasible, significantly enhances theoretical capacity, and reduces ion diffusion barriers, highlighting its potential as an anode material for alkali-ion batteries.^29^

However, despite these advances, a systematic and comparative investigation of how different substitutional dopants—B, P, and S, with their distinct electronegativities, atomic radii, and polarizabilities—and their concentrations modulate the structural stability, electronic structure, lithium adsorption behavior, diffusion kinetics, and voltage characteristics of C_2_N remains lacking. In particular, carbon-site substitution directly perturbs the π-conjugated backbone of the N-rich C_2_N lattice, generating C–dopant–N motifs that may critically influence charge transfer, Li binding strength, and electronic conductivity. A comprehensive understanding of these effects is essential for rational anode design.

In this work, a systematic first-principles density functional theory (DFT) study is performed to evaluate B-, P-, and S-doped C_2_N monolayers as lithium-ion battery anodes. Two dopant concentrations (2.78 and 5.56 at. %) are considered within a C_48_N_24_ supercell to elucidate concentration-dependent effects. Structural stability, electronic properties, lithium adsorption energetics, diffusion pathways, and open-circuit voltage profiles are comprehensively analyzed. This study establishes a comparative framework linking dopant chemistry and concentration to electrochemical performance, providing fundamental insights and rational design guidelines for high-capacity, fast-charging C_2_N-based anode materials.

## Computational Methods

DFT calculations were performed using the Vienna Ab Initio Simulation Package (VASP) with the projector-augmented wave (PAW) method. The exchange-correlation potential was described using the Perdew–Burke–Ernzerhof (PBE) functional within the generalized gradient approximation (GGA). A plane-wave cutoff energy of 500 eV was employed in all calculations. Long-range van der Waals interactions were included using the DFT-D3 method with Becke–Johnson damping.^30^ Structural optimizations were performed using the conjugate gradient method, with convergence criteria requiring atomic forces to be less than 0.01 eV/Å and total energy changes below 1 × 10^–5^ eV. A 2 × 2 × 1 supercell of the C_2_N monolayer, containing 48 C and 24 N atoms, was employed. A vacuum spacing of 18 Å prevented interlayer interactions. The Brillouin zone was sampled using a 5 × 5 × 1 Monkhorst–Pack grid for relaxation and a 9 × 9 × 1 grid for density of states (DOS) calculations.

The lithium adsorption energy was computed as

1 $$E_{\mathrm{ads}}=\frac{E_{{\mathrm{mLi}}_{x}}-E_{m}-n\mu_{\mathrm{Li}}}{n}$$

where *E*_mLi_*x*__ and *E*_m_ are the total energies of the Li-adsorbed and pristine monolayers, respectively, μ_Li_ is the chemical potential of Li in bulk Li metal, and *n* is the number of Li atoms.

The open-circuit voltage (OCV) was calculated between different lithiation stages as

2 $$\mathrm{OCV}=-\frac{E_{{\mathrm{Li}}_{x_{2}}}-E_{{\mathrm{Li}}_{x_{1}}}-\left(x_{2}-x_{1}\right)\mu_{\mathrm{Li}}}{\left(x_{2}-x_{1}\right)e}$$

where *x*_1_ and *x*_2_ are the lithium concentrations in adjacent configurations.

Li-ion diffusion barriers were computed using the climbing-image nudged elastic band (CI-NEB) method.^31^ Five intermediate images were generated between initial and final states to capture the minimum energy path. All other computational settings and parameters were kept consistent with those used in the relaxation calculations.

Ab initio molecular dynamics (AIMD) simulations were carried out in the canonical (NVT) ensemble using a Nosé–Hoover thermostat at 300 K for 10 ps with a 1 fs time step, to examine the thermal stability of both pristine and doped C_2_N monolayers.

Visualization of charge density, electron localization function (ELF), and atomic structures was performed using the VESTA package.^32^

## Results and Discussion

### X_2_-Doped C_2_N Monolayers (X = B, P, S)

#### Structural, Mechanical, and Thermal Properties

As illustrated in Figure 1, the unit cell of pristine C_2_N consists of 12 carbon (C) atoms and 6 nitrogen (N) atoms. This material exhibits semiconducting behavior, with a direct band gap of 1.66 eV calculated using the PBE functional (Figure S1). This value underestimates the experimental gap of 1.96 eV,^7^ while the HSE06 functional yields an improved value of 2.45 eV, in line with previous studies.^28^ To study the effects of B, P, and S doping, a 72-atom C_2_N supercell (C_48_N_24_) was used, in which two carbon atoms were substituted with dopants, corresponding to a concentration of 2.78 at. %, with one doped pore followed by one undoped pore.

**Figure 1:**
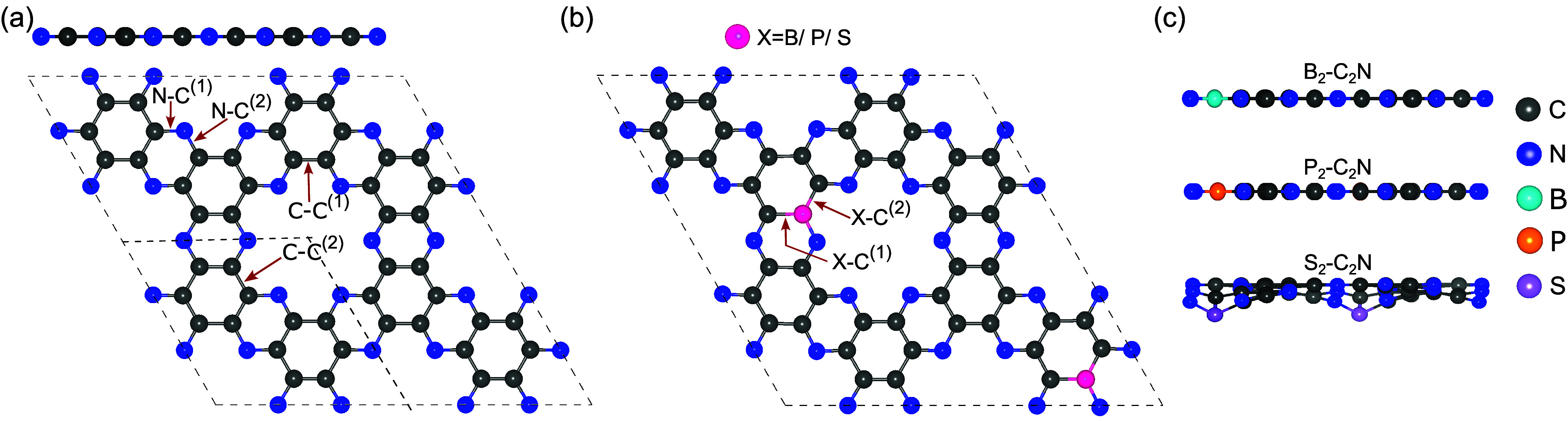
(a) Top and side views of pristine C_2_N. (b) Top view of X_2_–C_2_N (X = B, P, S). (c) Side views of B_2_–C_2_N, P_2_–C_2_N, and S_2_–C_2_N. Dark gray, blue, turquoise, orange, and purple spheres represent carbon (C), nitrogen (N), boron (B), phosphorus (P), and sulfur (S) atoms, respectively.

The atomic structures of pristine and X_2_-doped C_2_N (X = B, P, S) are presented in Figure 1 as representative models. Dopant incorporation effectively closes the band gap, leading to metallic behavior and significantly enhanced electronic conductivity. The optimized lattice constant of pristine C_2_N is 8.32 Å, consistent with literature values. The calculated bond lengths are summarized in Table 1. Upon doping, notable changes appear in the local bonding environment: C–C bonds contract slightly, while dopant–C and dopant–N bonds elongate. Among the dopants, B induces the least distortion, whereas P and S lead to more pronounced variations. This trend can be attributed to their larger atomic radii—B (0.81 Å), P (0.99 Å), and S (0.87 Å)—compared to those of C (0.65 Å) and N (0.54 Å). The size mismatch introduces local strain and geometric rearrangements around dopant sites.

**Table 1:** Comparison of Bond Lengths (Å) between Pristine and X_2_-C_2_N (X = B, P, S)

Structure	C–C^(1)^	C–C^(2)^	N–C^(1)^	N–C^(2)^	X–C^(1)^	X–C^(2)^	X–N
C_2_N	1.47	1.43	1.34	1.33	–	–	–
B_2_–C_2_N	1.44	1.41	1.33	1.32	1.54	1.51	1.37
P_2_–C_2_N	1.41	1.41	1.31	1.34	1.67	1.64	1.55
S_2_–C_2_N	1.44	1.41	1.34	1.32	1.68	1.63	1.55
ref 19	1.47	1.43	1.34	–	–	–	–

Defect formation energies were calculated to evaluate the thermodynamic feasibility of dopant incorporation into the C_2_N monolayer. The defect formation energy *E*_*f*_ is defined as *E*_*f*_ = *E*_doped_ – *E*_C_2*N*__ – ∑_*i*_ *n*_*i*_^add^ μ_*i*_^add^ + ∑_*j*_ *n*_*j*_^rem^ μ_*j*_^rem^, where *n*_*i*_^add^ (*n*_*j*_^rem^) are the number of atoms added and removed, respectively. The calculated formation energies indicate that B doping is the most favorable, with *E*_*f*_ = 1.36 eV, while P and S dopants exhibit higher formation energies of 5.36 and 4.40 eV, respectively. The relatively low formation energy for B suggests that its incorporation is thermodynamically feasible under typical high-temperature growth conditions, whereas the high formation energies of P and S imply very low equilibrium solubility. The equilibrium dopant concentration can be estimated using Boltzmann statistics: $c\approx \exp\left(-\frac{E_{f}}{k_{B}T}\right)$ . At a typical growth temperature of 1000 K (*k*_*B*_ *T* ≈ 86 meV), this gives an estimated dopant concentration of approximately 1.4 × 10^–7^ for B, 2.8 × 10^–28^ for P, and 7.2 × 10^–23^ for S. These results indicate that B doping is achievable under equilibrium growth conditions, whereas P and S dopants would require nonequilibrium synthesis methods (such as chemical vapor deposition or plasma-assisted growth) to be incorporated.

To evaluate the mechanical robustness of the doped systems, the elastic stability criteria for 2D materials were examined.^33^ For a mechanically stable 2D structure, the requirements *C*_11_ > 0, *C*_11_ *C*_22_ > *C*_12_^2^, and det­(*C*_*ij*_) > 0 must be satisfied. Based on the calculated elastic constants (Table 2), all pristine and X_2_–C_2_N (X = B, P, S) monolayers fulfill these conditions, confirming their mechanical stability. Pristine C_2_N exhibits high in-plane stiffness with *C*_11_ = 161.84 N/m and a Young’s modulus of 149.59 N/m, indicating strong mechanical rigidity. Upon doping, a moderate reduction in stiffness is observed. B_2_–C_2_N and P_2_–C_2_N display similar elastic responses, with Young’s moduli of 139.07 and 140.42 N/m, respectively, suggesting that B and P incorporation only slightly softens the lattice. In contrast, S_2_–C_2_N shows a pronounced decrease in stiffness, with *C*_11_ = 118.76 N/m and a significantly lower Young’s modulus of 90.66 N/m, indicating considerable lattice softening. The shear modulus follows the same trend, decreasing from 58.66 N/m for pristine C_2_N to approximately 53–55 N/m for B- and P-doped systems, and further down to 25.38 N/m for S doping. Additionally, the Poisson’s ratio slightly increases for B- and P-doped systems (∼ 0.28–0.29) and rises markedly for S_2_–C_2_N (ν = 0.65), implying enhanced transverse deformation and increased structural flexibility. Overall, although dopant incorporation reduces the intrinsic stiffness of C_2_N to varying degrees, all X_2_–C_2_N monolayers remain mechanically stable. B and P dopants largely preserve the mechanical integrity of the lattice, whereas S doping significantly weakens the structure while maintaining elastic stability.

**Table 2:** Calculated Elastic Constants (*C*_*ij*_), Young’s Modulus (*E*), Shear Modulus (*G*), and Poisson’s Ratio (*ν*) of Pristine and X_2_-C_2_N (X = B, P, S) Monolayers

System	*C*_11_ (N/m)	*C*_22_ (N/m)	*C*_12_ (N/m)	*C*_66_ (N/m)	*C*_16_ (N/m)	*C*_26_ (N/m)	*E* (N/m)	*G* (N/m)	ν
C_2_N	161.84	—	44.52	—	—	—	149.59	58.66	0.27
B_2_–C_2_N	148.27	152.58	47.94	55.80	–1.50	2.16	139.07	53.45	0.29
P_2_–C_2_N	148.94	152.30	44.80	56.66	–2.28	–0.49	140.42	54.75	0.28
S_2_–C_2_N	118.76	72.82	25.16	51.21	0.18	–38.24	90.66	25.38	0.65

The electron localization function (ELF) maps in Figure 2(a–d) provide clear insight into the bonding characteristics of the systems. ELF values of 1.0, 0.5, and 0.0 correspond to perfectly localized electrons (covalent bonding), delocalized electrons (metallic bonding), and low electron localization (electron depletion/ionic character), respectively. In pristine C_2_N, ELF values approaching 1 are concentrated along C–C bonds, indicative of strong covalent interactions. Around N atoms, pronounced ELF localization highlights partial electron confinement and lone-pair character, consistent with a mixed covalent–ionic bonding environment. In doped C_2_N, enhanced ELF localization appears along dopant–C and dopant–N bonds, evidencing robust covalent integration of B, P, and S atoms into the lattice. Although these dopants differ in valence electron configuration and electronegativity, their incorporation does not disrupt the intrinsic bonding topology of the C_2_N framework. The extended C–C and C–N covalent framework remains intact, with only localized electronic redistribution around the dopant sites, demonstrating that substitution induces subtle electronic modulation rather than structural destabilization of the monolayer.

**Figure 2:**
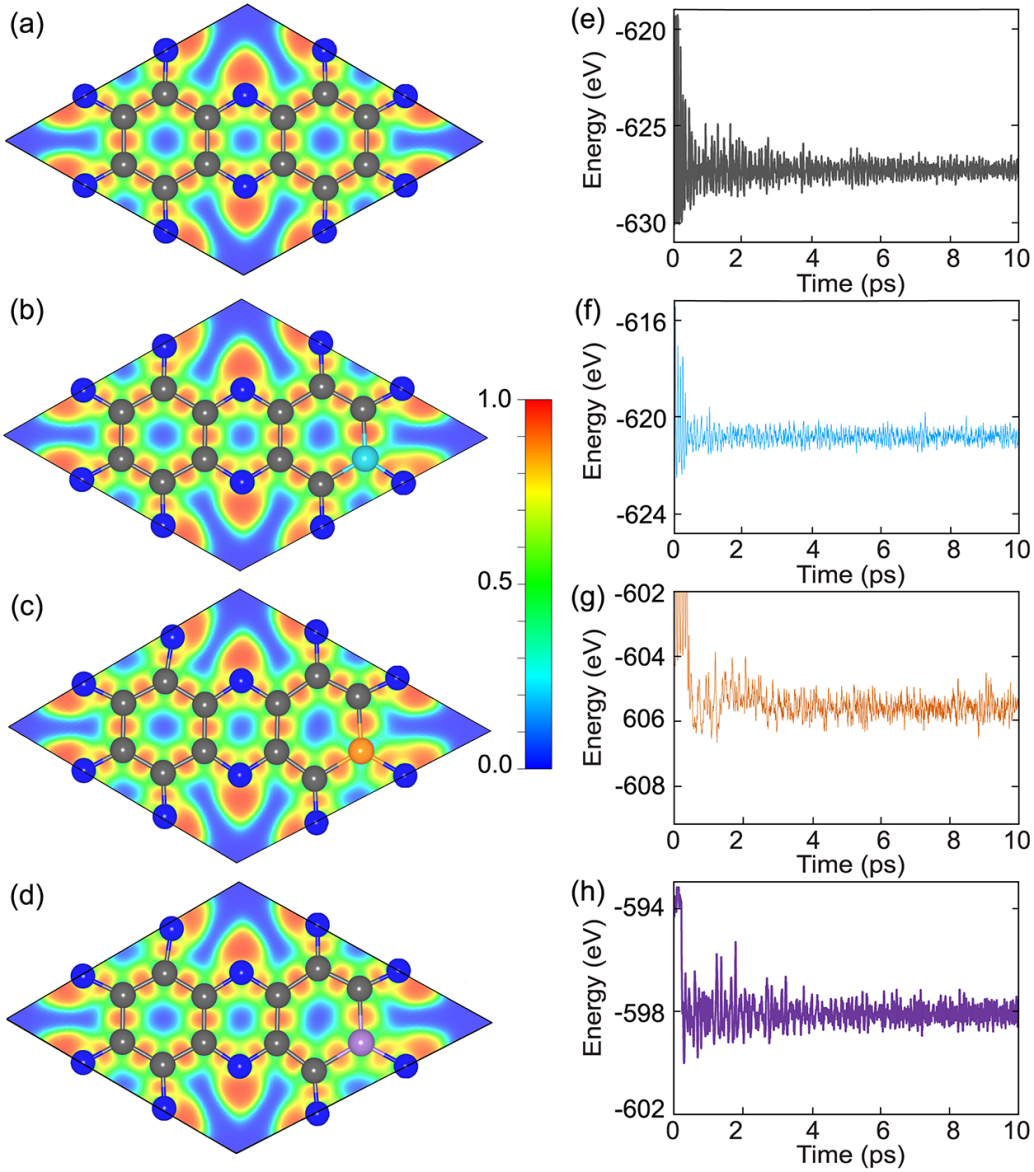
(a–d) Electron Localization Function (ELF) maps for pristine C_2_N, B_2_–C_2_N, P_2_–C_2_N, and S_2_–C_2_N monolayers, respectively. (e–h) Total energy profiles from ab initio molecular dynamics (AIMD) simulations at 300 K over a 10 ps time span for the same systems, demonstrating their thermal stability.

Thermal stability was further examined using AIMD simulations at 300 K for 10 ps (Figure 2e–h). In both pristine and doped systems, the total energy fluctuates around a stable average without sharp increases or decreases, demonstrating robust structural stability at room temperature. The doped system exhibits slightly larger oscillations than the pristine case, likely due to strain and mass differences introduced by dopant atoms. However, no abrupt instabilities are observed.

#### Li Adsorption, Electronic Structure, and Electrochemical Performance

For a material to be viable as an anode in lithium-ion batteries, it must meet several key requirements: (i) high lithium storage capacity, (ii) negative adsorption energies at all concentrations, (iii) absence of lithium protrusion from the surface, and (iv) reversible structural behavior during lithiation and delithiation. To assess the performance of pristine and X_2_-C_2_N monolayers, Li atoms were adsorbed at various potential sites (Figure 3 and Table 3). In pristine C_2_N and B_2_–C_2_N, Li preferentially occupies the hollow (H) site at the center of the pore, even when initially placed on cavity (h_1_, h_2_, h_3_) or bridge (B) positions. The nearby cavity sites exhibit adsorption energies comparable to H, resulting in only a modest driving force for Li relocation during relaxation. In contrast, P and S doping significantly deepen the energy minimum at the hollow site, rendering the pore center substantially more favorable than the cavity positions. Consequently, Li spontaneously migrates toward the hollow site in P_2_–C_2_N and S_2_–C_2_N, regardless of its initial adsorption location.

**Figure 3:**
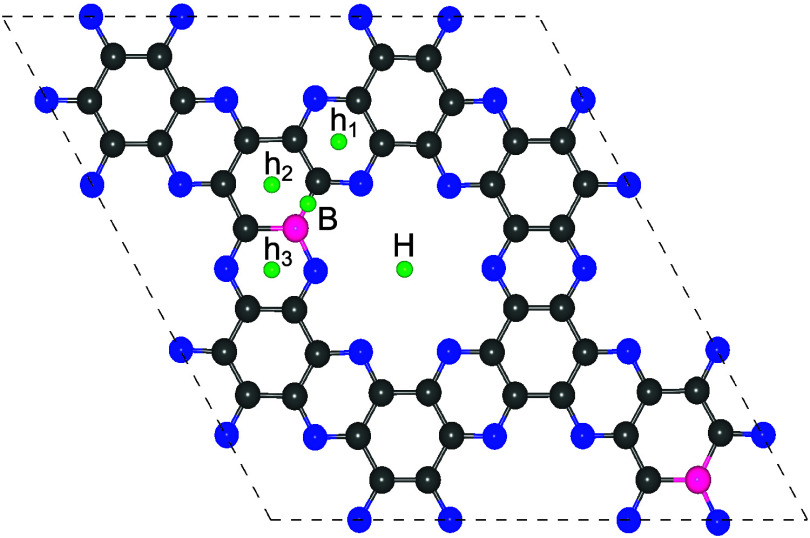
Various possible adsorption configurations of a single Li atom on pristine and X_2_-C_2_N (X = B, P, S). The H site corresponds to a hollow position, h_1_–h_3_ denote cavity sites, and B represents a bridge site.

**Table 3:** Adsorption Energies at Different Adsorption Sites on Pristine and X_2_-C_2_N (X = B, P, S)

Structure	Site	*E*_ads_ (eV)
C_2_N	H → H	–2.60
	h_1_ → h_1_	+0.15
	h_2_ → h_2_	+0.36
	h_3_ → h_3_	+0.15
	B → H	–2.70
B_2_–C_2_N	H → H	–3.94
	h_1_ → h_1_	–1.07
	h_2_ → h_2_	–1.13
	h_3_ → h_3_	–1.50
	B → H	–4.09
P_2_–C_2_N	H → H	–4.85
	h_1_ → H	–4.97
	h_2_ → h_2_	–2.12
	h_3_ → H	–5.21
	B → H	–5.08
S_2_–C_2_N	H → H	–2.66
	h_1_ → H	–2.62
	h_2_ → H	–2.63
	h_3_ → h_3_	–0.27
	B → H	–2.63

Figure 4 shows optimized adsorption structures for pristine C_2_N and S_2_–C_2_N, while Figure S2 presents B_2_–C_2_N and P_2_–C_2_N at varying Li concentrations (Li_0.05_, Li_0.10_, Li_0.16_), which show that Li adsorption clearly depends on both concentration and doping. Only minor distortions are observed, mainly localized near adsorption sites, while the overall lattice remains intact, indicating mechanical robustness under lithiation. For pristine C_2_N, Li atoms preferentially occupy hollow or cavity sites, and as the Li concentration increases, adsorption gradually extends to less favorable sites due to inter-Li interactions, while the overall lattice framework remains intact. A similar trend is observed in X_2_–C_2_N, where doping enhances Li binding at low coverage, likely through charge redistribution and local polarization induced by the dopant. Even at higher Li concentrations, although binding weakens due to Li–Li interactions, structural distortions remain minor and localized, demonstrating the lattice’s robustness during lithiation. To further evaluate structural robustness upon lithiation, the optimized cell volumes were examined at different Li concentrations. The calculated volume variations are negligible (<0.002%), indicating that Li insertion does not induce noticeable lattice expansion. This confirms the excellent structural rigidity of pristine and doped C_2_N systems and suggests minimal mechanical degradation during charge–discharge processes, even at high Li concentrations.

**Figure 4:**
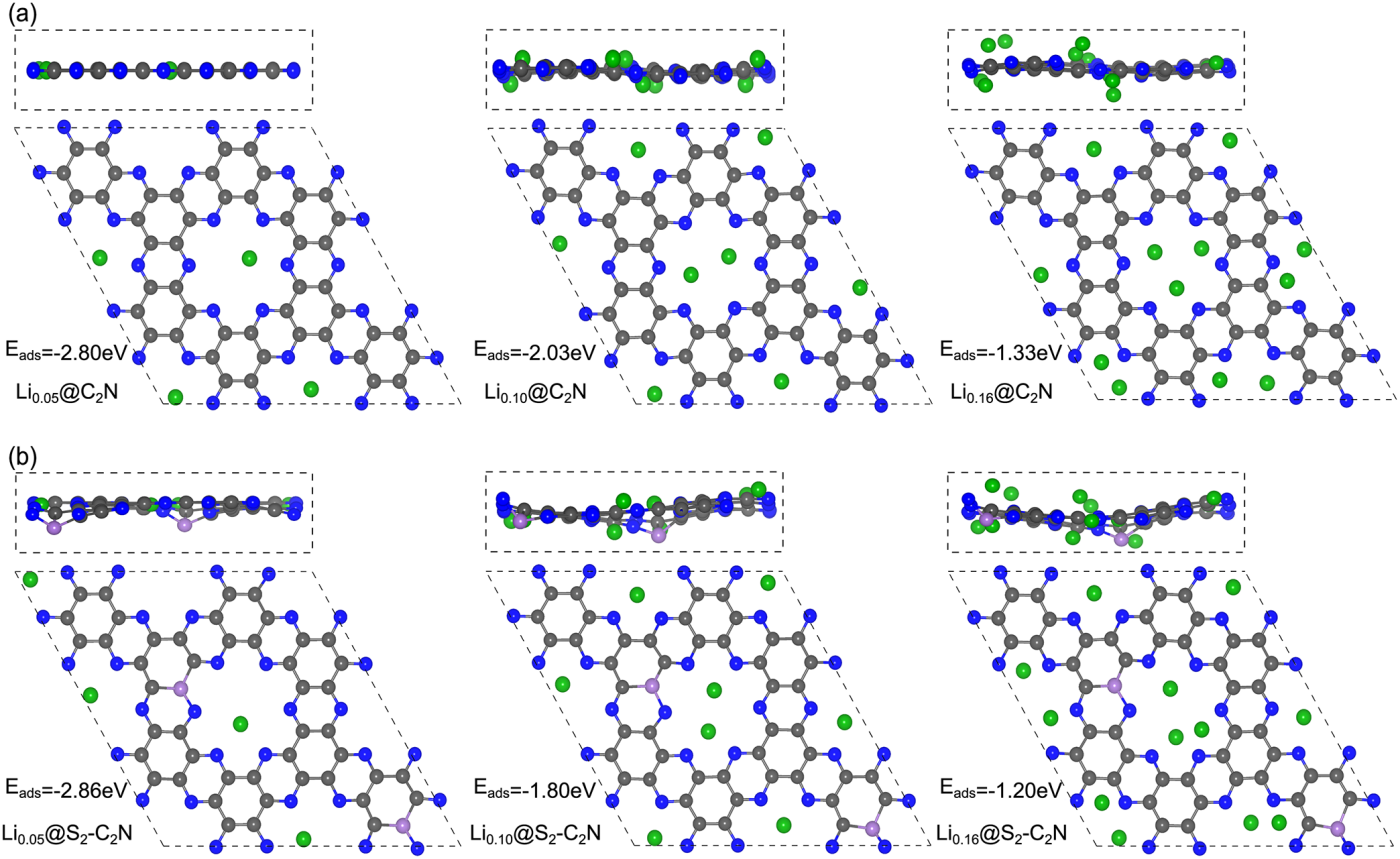
Top and side views of optimized Li adsorption structures on (a) pristine C_2_N and (b) S_2_–C_2_N at varying concentrations. The corresponding adsorption energies (*E*_ads_) are indicated for each configuration.

To further assess the thermal stability of the lithiated systems, additional AIMD simulations were performed for the structures with the highest Li concentration at 300 K for 10 ps (Figure 5). The fully lithiated configurations exhibit stable energy fluctuations around an equilibrium value without any abrupt structural distortions or bond–breaking events. The Li atoms remain adsorbed at their preferred sites throughout the simulation, confirming the robustness of the predicted adsorption configurations at finite temperature.

**Figure 5:**
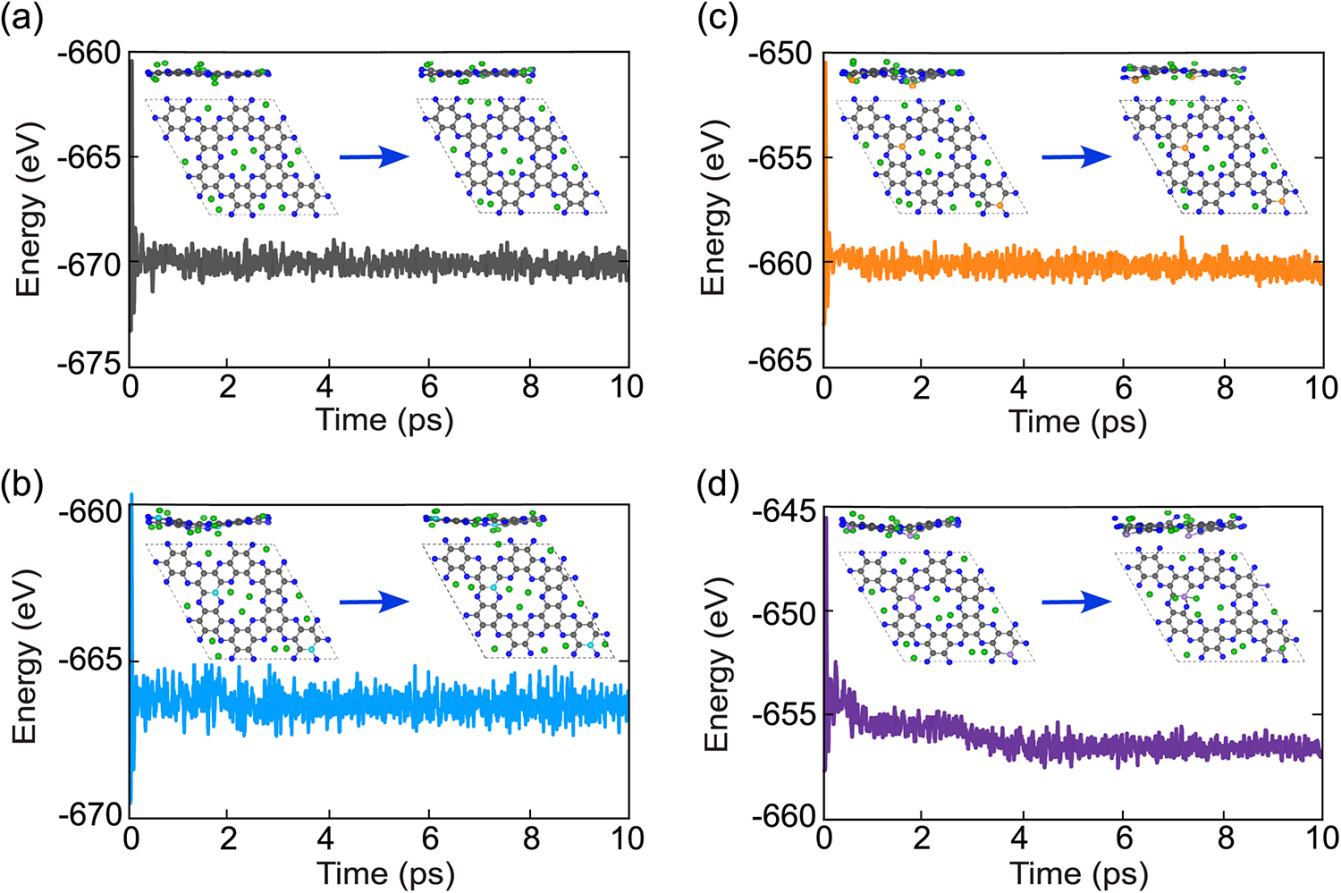
(a–d) AIMD results for pristine C_2_N, B_2_–C_2_N, P_2_–C_2_N, and S_2_–C_2_N monolayers at the highest Li concentration (Li_0.16_) at 300 K for 10 ps.

Adsorption energies as a function of Li concentration are shown in Figure 6. All systems exhibit negative adsorption energies across the studied range, confirming thermodynamically favorable Li uptake. Among the doped structures, P_2_–C_2_N and B_2_–C_2_N display stronger affinity toward Li than pristine C_2_N, especially at low concentrations. In particular, P_2_–C_2_N demonstrates the strongest binding in the dilute limit. Meanwhile, S_2_–C_2_N achieves a desirable compromise between strong adsorption and minimal structural distortion, making it promising where both electrochemical performance and stability are critical. As Li concentration increases, adsorption energies gradually become less negative due to enhanced Li–Li repulsion, a trend consistently observed across all systems.

**Figure 6:**
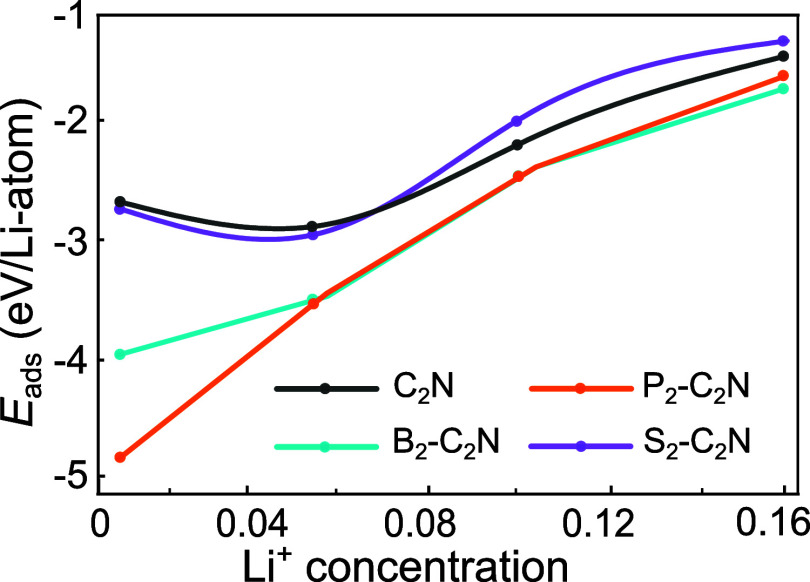
Adsorption energy as a function of Li concentration for pristine and X_2_-C_2_N (X = B, P, S).

The projected density of states (PDOS) plots (Figures 7 and S3) further clarify the electronic effects of doping before and after lithiation. In pristine C_2_N, the Fermi level lies near the valence band maximum, confirming semiconducting behavior. B doping introduces 2p states just above the valence band, consistent with p-type doping, as B (Group 13) atoms accept electrons and generate holes in the valence band. In contrast, P (Group 15) and S (Group 16) doping contribute additional electrons, introducing donor states near the conduction band minimum. These n-type dopants shift the Fermi level upward. Notably, S doping introduces broader 3p states, which may form midgap states that act as deep traps or lead to metallic characteristics at higher concentrations.

**Figure 7:**
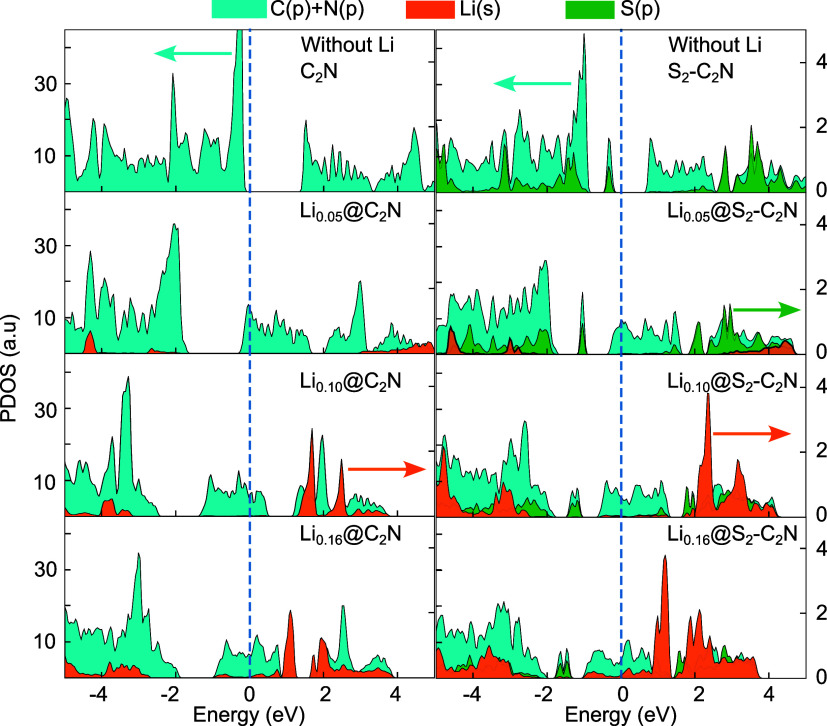
Projected density of states (PDOS) of pristine and S_2_–C_2_N before and after lithiation at various Li concentrations. The turquoise, orange, and green shaded regions represent the contributions from C­(*p*)+N­(*p*), Li­(*s*), and S­(*p*), respectively. The turquoise arrow points to the left *y*-axis, which corresponds to the PDOS of C­(*p*)+N­(*p*), whereas the orange and green arrows refer to the right *y*-axis, representing the PDOS of Li­(*s*) and S­(*p*), respectively.

Upon lithiation, the Fermi level shifts upward across all systems, consistent with electron injection from Li atoms. Li s-states appear near or just below the conduction band minimum, reflecting electron donation to the substrate. While Bader charge analysis^34^ indicates that the total transferred charge from the Li atoms to the substrate is substantial and nearly identical across pristine and doped systems (∼0.9 |*e*|, 3.65 |*e*|, 6.96 |*e*|, and 10.37 |*e*| for 1, 4, 8, and 12 Li atoms, respectively), PDOS analysis reveals subtle differences in the electronic structure due to dopant effects. In particular, partial overlap between Li s-states and P 3p or S 3p states at higher Li concentrations indicates dopant-induced orbital polarization, which can enhance electronic conductivity, even though the Li–substrate interaction remains predominantly ionic.

Figure 8 shows the OCV profiles of pristine and X_2_-C_2_N as a function of Li concentration (up to 0.16). All systems exhibit a stepwise voltage drop due to Li–Li repulsion. The calculated average OCV values follow the order P_2_–C_2_N (≈ 2.93 V) > B_2_–C_2_N (≈ 2.77 V) > pristine C_2_N (≈ 2.18 V) > S_2_–C_2_N (≈ 2.12 V). Among the studied systems, P_2_–C_2_N and B_2_–C_2_N deliver much higher charging voltages compared with many existing anode materials and are comparable to several reported 2D candidates such as α-SiN (2.26 V),^35^ α-SiP (2.14 V),^35^ b-P (2.9 V),^36^ SnS,^37^ and SnSe (2.22 V).^37^ However, their voltages remain lower than those of some high-voltage 2D materials such as b-As (3.5 V)^38^ and MoN_2_ (3.64 V).^39^ On the other hand, pristine C_2_N and S_2_–C_2_N exhibit significantly lower average voltages relative to the P- and B-doped systems. Their values are close to that of the commercial TiO_2_ anode (∼ 1.5–1.8 V),^40^ indicating that pristine and S-doped C_2_N offer favorable low-voltage characteristics desirable for practical anode operation.

**Figure 8:**
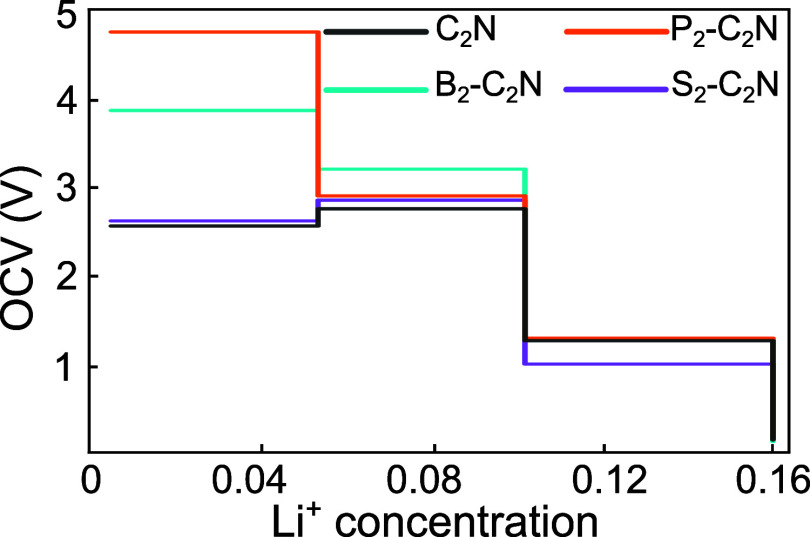
Open-circuit voltage (OCV) profiles as a function of Li concentration for pristine and X_2_-C_2_N (X = B, P, S).

#### Lithium Diffusion Pathways and Energy Barriers

Efficient Li diffusion is crucial for achieving high rate capability in anode materials. To explore the migration behavior of Li in X_2_-C_2_N, we performed climbing-image nudged elastic band (CI-NEB) calculations. Two distinct scenarios were considered: (i) Li migration near dopant sites (Figure 9a,c) and (ii) Li migration between neighboring doped and undoped pores across a carbon-rich bridge ring (Figure 9b,d). These two cases highlight the influence of local chemical environments and dopant proximity on diffusion energetics.

**Figure 9:**
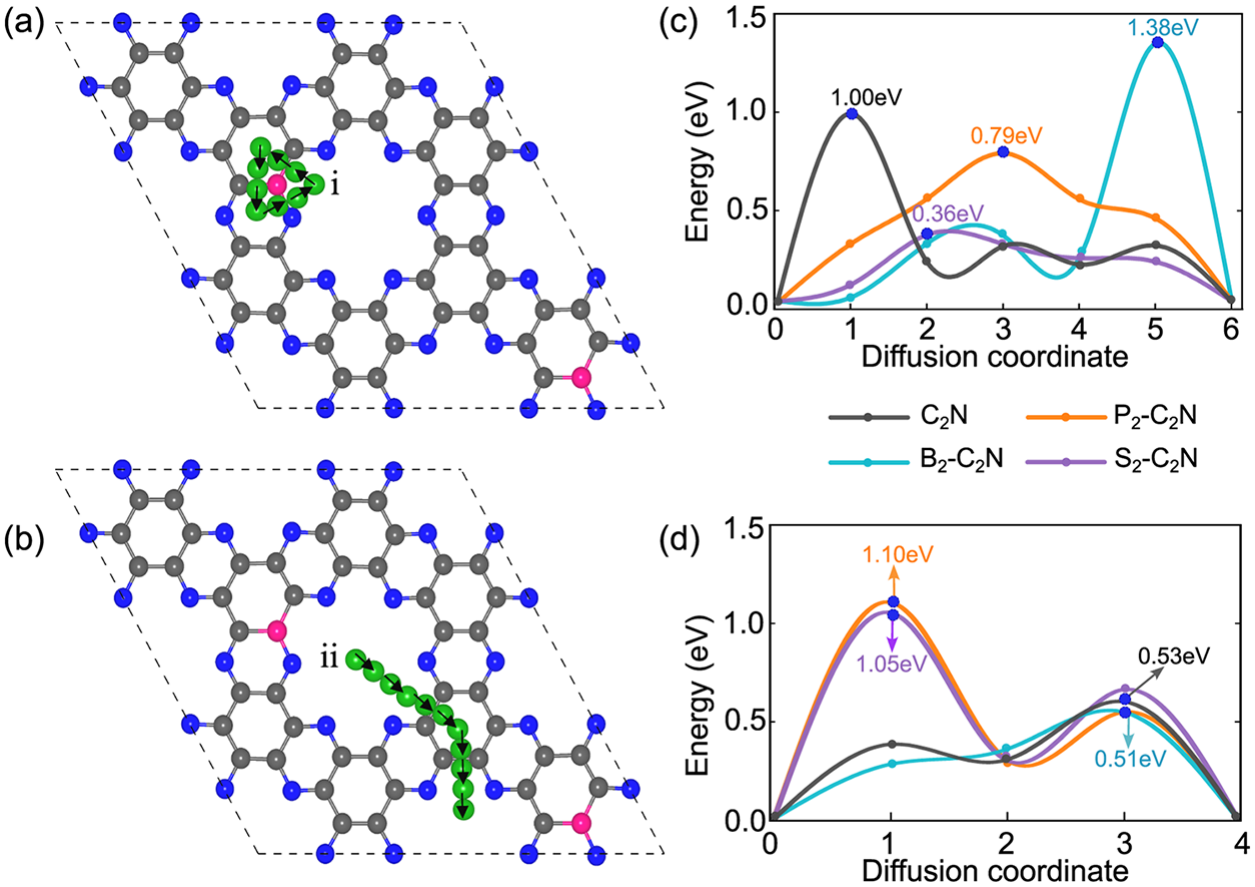
Li migration pathways and diffusion energy profiles in X_2_-C_2_N: (a, c) migration near dopant sites and (b, d) migration across undoped pores via carbon-rich bridge rings. The blue markers denote the maximum diffusion barriers for pristine and X_2_-C_2_N (X = B, P, S).

For B_2_–C_2_N, a deep energy well forms when Li is positioned near the B dopant. This strong stabilization produces a high diffusion barrier of ∼1.4 eV, substantially higher than that of pristine C_2_N. The electron-deficient nature of B atoms, which act as Lewis acidic sites, strongly attracts Li^+^ ions, creating potential traps that hinder Li migration. However, when Li moves away from the doped pore toward an undoped region (Figure 9d), the barrier decreases significantly to ∼0.5 eV, suggesting that Li mobility improves once it escapes the influence of the dopant.

In the case of P_2_–C_2_N, the energy landscape shows a diffusion barrier of ∼0.8 eV near the dopant (Figure 9c) and ∼1.1 eV far from the dopant (Figure 9d). This indicates a relatively uniform interaction strength along the migration pathway. The relatively high and consistent barriers suggest that Li mobility may be limited, which could affect the rate capability of P-doped C_2_N as an anode material.

For S_2_–C_2_N, a distinct behavior is observed. Near the S dopant, Li is only weakly stabilized, with a diffusion barrier of ≈ 0.36 eV (Figure 9c). This relatively weak interaction may arise from the diffuse and weakly directional nature of chalcogen–cation interactions, which are less effective at stabilizing small cations such as Li^+^. As Li migrates from the S-doped pore toward the carbon-bridge ring, steric and electrostatic constraints along the pathway increase the diffusion barrier to ≈ 1.05 eV (Figure 9d). This observation indicates that Li favors regions of the C_2_N framework that are more electrostatically neutral and geometrically relaxed.

Overall, these results highlight the strong dependence of Li mobility on dopant type. While B doping introduces deep potential traps that hinder diffusion, P doping yields moderate and relatively uniform barriers, and S doping enables a balance of weak dopant binding and accessible migration barriers. This makes S_2_–C_2_N particularly attractive for applications requiring fast ion transport.

### Highly Doped X_4_-C_2_N Monolayers (X = B, P, S)

#### Structural and Electronic Properties

Figure 10 shows the optimized structures of X_4_–C_2_N (X = B, P, S), representing the highly doped systems with a 4-dopant configuration corresponding to 5.56 at. %, in which all nanopores are doped. Compared to the S_2_–C_2_N monolayers, the S_4_–C_2_N structures appear significantly flatter, while the B_4_–C_2_N and P_4_–C_2_N structures remain largely unchanged, as both were already planar in the X_2_ systems. The increased planarity in S_4_–C_2_N can be attributed to the higher dopant concentration, which more evenly distributes strain across the lattice and reduces local distortions near the dopant sites.

**Figure 10:**
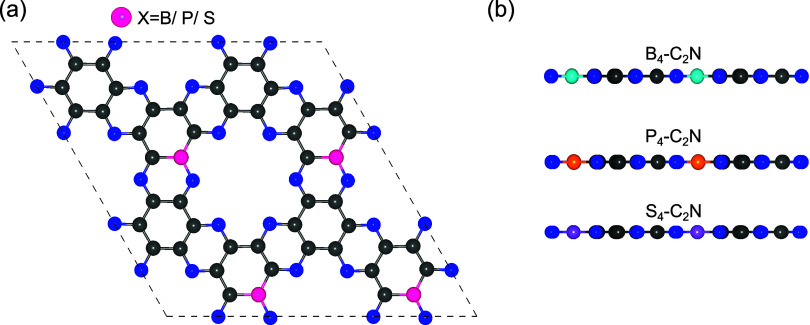
(a) Top view of X_4_–C_2_N (X = B, P, S). (b) Side views of B_4_–C_2_N, P_4_–C_2_N, and S_4_–C_2_N. Dark gray, blue, turquoise, orange, and purple spheres represent carbon (C), nitrogen (N), boron (B), phosphorus (P), and sulfur (S) atoms, respectively.

Figure S5 presents the PDOS of the X_4_–C_2_N systems at different lithiation levels. Compared with the corresponding X_2_–C_2_N structures, X_4_–C_2_N exhibits stronger orbital overlap and enhanced metallization between the *p* states of C/N and the dopant atoms near the Fermi level. This enhanced hybridization indicates stronger electronic coupling between Li and the host framework, facilitating charge transfer and potentially improving electronic conductivity. These strong local interactions around dopant sites may contribute to increased diffusion barriers and overpotentials, potentially limiting Li mobility and rate performance.

#### Li Adsorption and Diffusion

Figures S4 summarize the optimized structures of X_4_–C_2_N at varying Li concentrations (Li_0.05_, Li_0.16_, Li_0.27_). Li atoms preferentially occupy hollow or cavity sites, and as the Li concentration increases, inter-Li repulsion drives adsorption onto less favorable positions within the lattice. Notably, after the initial stage of lithiation, X_4_–C_2_N monolayers show more pronounced structural distortions and stronger Li adsorption energies compared to the corresponding X_2_–C_2_N systems, indicating that Li adsorption is strongly enhanced by higher dopant concentrations. Higher dopant densities modify the local electronic environment and lattice strain, increasing structural flexibility and allowing Li atoms to occupy additional sites, which increases the total Li storage capacity, even though the average adsorption energy per Li atom decreases with increasing Li coverage due to site saturation and Li–Li repulsion. For the X_4_–C_2_N systems, the adsorption energy ranges are −4.09 eV to −1.01 eV for B_4_–C_2_N, −3.98 eV to −1.10 eV for P_4_–C_2_N, and −4.55 eV to −1.11 eV for S_4_–C_2_N, corresponding to Li coverages from Li_0.05_ to Li_0.27_.

To clarify the migration behavior, CI-NEB calculations reveal two contrasting trends in X_4_–C_2_N: (i) Li migration barriers near dopant clusters (Figure S6­(a,c)) are higher than in X_2_–C_2_N, indicating stronger trapping effects; and (ii) Li migration between neighboring doped pores across carbon-rich bridge rings (Figure S6­(b,d)) is relatively reduced due to partial charge delocalization. However, the deep potential wells formed around high dopant concentrations create strong Li trapping sites, which ultimately hinder overall migration. These results demonstrate a clear trade-off: while high dopant concentrations enhance Li storage capacity and binding strength, they introduce diffusion anisotropy and trapping effects that may compromise rate performance. This balance highlights the importance of carefully tuning substitutional doping levels to optimize both capacity and Li transport for practical applications.

This study provides a systematic analysis of individual dopants on C_2_N monolayers, offering insights into their influence on structural, electronic, and electrochemical properties. Nevertheless, codoping—particularly B/S codoping—represents an intriguing avenue for future research.^41,42^ The combination of an electron-deficient dopant (B) with a more electronegative element (S) may induce synergistic electronic effects, potentially altering charge distribution and band structure. Such effects could further modulate adsorption behavior and enhance electrochemical performance, warranting dedicated investigation in future studies.

## Conclusions

This work systematically explores the effects of B, P, and S doping on C_2_N monolayers for lithium-ion battery anodes. B doping is thermodynamically most feasible under equilibrium growth, whereas P and S require nonequilibrium methods. All doped monolayers remain mechanically stable, with S doping significantly softening the lattice, introducing structural flexibility that enables fast Li diffusion with a low barrier of ∼0.36 eV. P-, B-, and S-doped C_2_N exhibit average open-circuit voltages (OCV) of 2.93, 2.77, and 2.12 V, respectively, demonstrating that P and B doping provide high-voltage operation while S doping offers low-voltage, high-rate capability. High dopant concentrations (X_4_–C_2_N) increase Li storage capacity by enabling adsorption at additional sites (up to Li_0.27_ coverage) and stronger Li–host interactions (adsorption energies down to −4.55 eV), though at the cost of diffusion anisotropy and Li trapping. Among all systems, S-doped C_2_N achieves a unique combination of moderate adsorption, rapid Li mobility, and structural adaptability, balancing capacity, voltage, and rate performance. These results provide quantitative design principles for doped C_2_N anodes, and suggest that optimizing dopant type and concentration—or exploring codoping strategies—can simultaneously maximize Li storage, rate capability, and mechanical robustness, paving the way for high-performance, fast-charging 2D anodes.

## Supplementary Material



## Notes

Calculated Li adsorption energies, average open-circuit voltages, and diffusion barriers for pristine and doped C_2_N systems (Table S1); electronic band structure and charge density distributions of pristine C_2_N (Figure S1); optimized Li adsorption structures for B_2_–C_2_N, P_2_–C_2_N, B_4_–C_2_N, P_4_–C_2_N, and S_4_–C_2_N at different concentrations (Figures S2 and S4); projected density of states before and after lithiation (Figures S3 and S5); and Li migration pathways and diffusion energy profiles in doped C_2_N systems (Figure S6) (PDF)
