# Supplementary material for: First-Principles Insights into Li Storage and Ion Diffusion in B‑, P‑, and S‑Doped C2N Anodes
Source: ACS Omega. 2026 Apr 1;11(14):22007–16. doi: 10.1021/acsomega.5c12977 (PMC13084508; doi:10.1021/acsomega.5c12977)
Supplement: Supplementary file 1 [file ao5c12977_si_001.pdf]

Supporting Information for: First-Principles Insights into Li Storage and Ion Diffusion in B-, P-, and S-Doped C_2N Anodes

Fereshteh Ghorbani Shadpey[1], Maryam Soleimani[1], and Mahdi Pourfath*[1,2]

[1]School of Electrical and Computer Engineering, College of Engineering, University of Tehran, Tehran 14395-515, Iran
[2]Institute for Microelectronics/E360, TU Wien, A-1040 Vienna, Austria

*Emails: pourfath@ut.ac.ir; pourfath@iue.tuwien.ac.at

Table S1: Calculated Li adsorption energies (E_{ads}), average open-circuit voltages (OCV), and diffusion barriers for pristine and X_2-C_2N (X = B, P, S). E_{barrier1} corresponds to the highest migration barrier near dopant sites, and E_{barrier2} corresponds to migration across undoped pores via carbon-rich bridge rings. For comparison purposes, previously reported E_{ads}, average OCV, and diffusion barrier values of other anode materials are also listed.

Structure	E_{ads} (eV)	OCV (V)	E_{barrier1} (eV)	E_{barrier2} (eV)
C_2N	−2.60	2.18	1.00	0.53
B_2-C_2N	−3.94	2.77	1.38	0.51
P_2-C_2N	−4.85	2.93	0.79	1.10
S_2-C_2N	−2.66	2.12	0.36	1.05
Ref[1]	−2.95	2.37	0.71	–
Ref[2]	−0.99	0.43	0.10	–
Ref[3]	−3.36	3.36	–	–
Ref[4]	−2.58	0.47	0.46	–
Ref[5]	−0.85	1.13	0.60	–
Ref[6]	−2.00	1.00	0.64	–
Ref[7]	−2.70	1.60	0.50	–
Ref[8]	−3.35	1.32	0.81	–
Ref[9]	−2.50	1.30	–	–

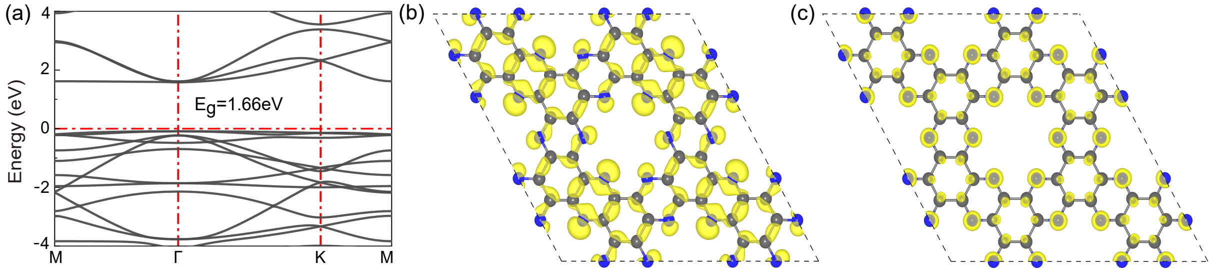

Figure S1: (a) Electronic band structure of monolayer C_2N calculated using the PBE functional. (b,c) Charge density distributions corresponding to the valence band maximum (VBM) and conduction band minimum (CBM) of pristine monolayer C_2N, showing that the frontier states are uniformly distributed over the C and N atoms. The delocalized frontier states in pristine C_2N indicate favorable electronic conductivity and efficient charge redistribution upon Li adsorption, which are desirable features for anode materials.

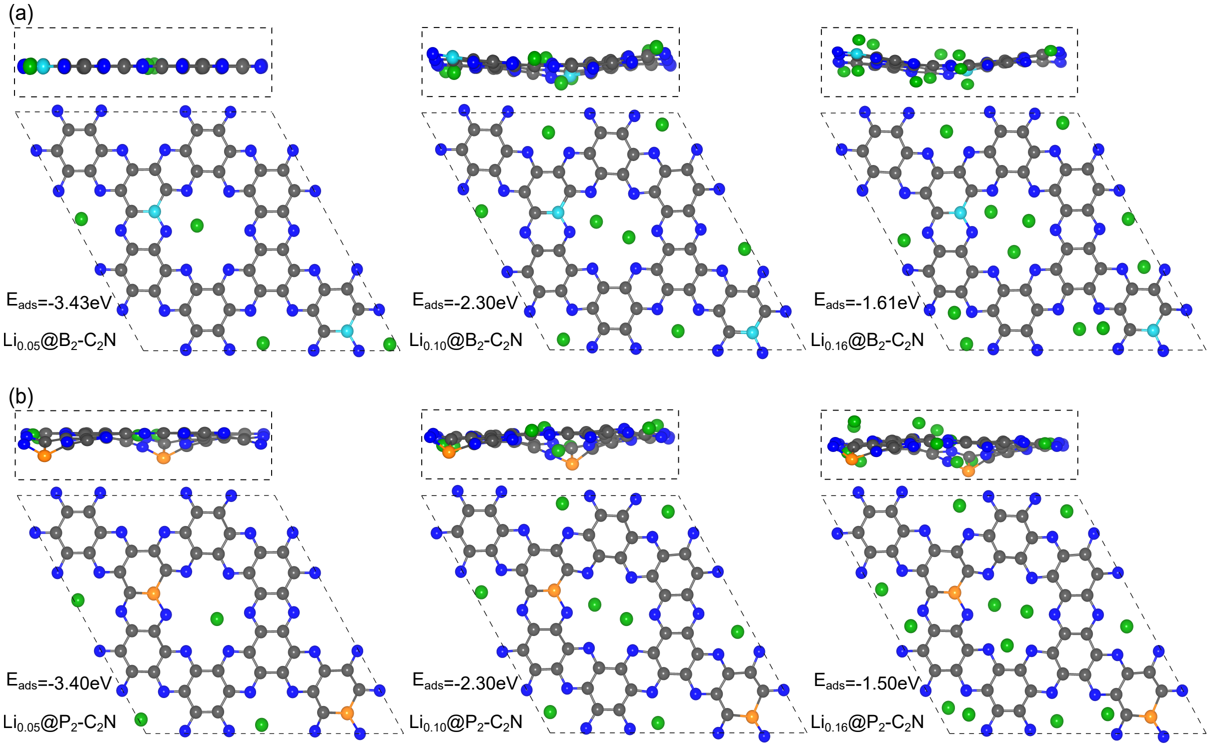

Figure S2: Top and side views of optimized Li adsorption structures on (a) B_2-C_2N and (b) P_2-C_2N at varying concentrations. The corresponding adsorption energies (E_{ads}) are indicated for each configuration.

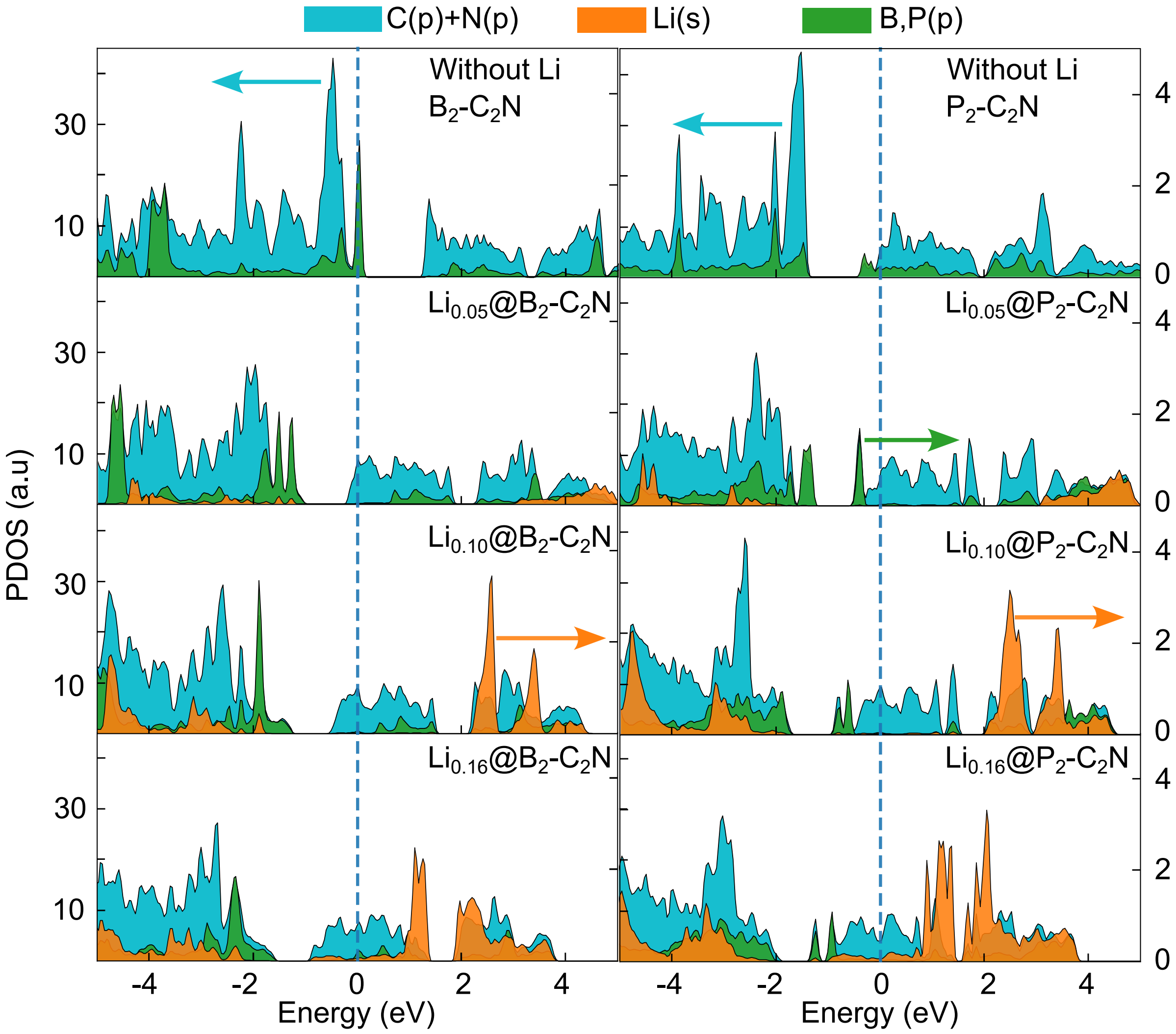

Figure S3: Projected density of states (PDOS) of B_2-C_2N and P_2-C_2N before and after lithiation at different concentrations. The turquoise, orange, and green shaded regions represent the contributions from C(p)+N(p), Li(s), and B, P(p), respectively. The turquoise arrow points to the left y-axis, which corresponds to the PDOS of C(p)+N(p), whereas the orange and green arrows refer to the right y-axis, representing the PDOS of Li(s) and B, P(p), respectively.

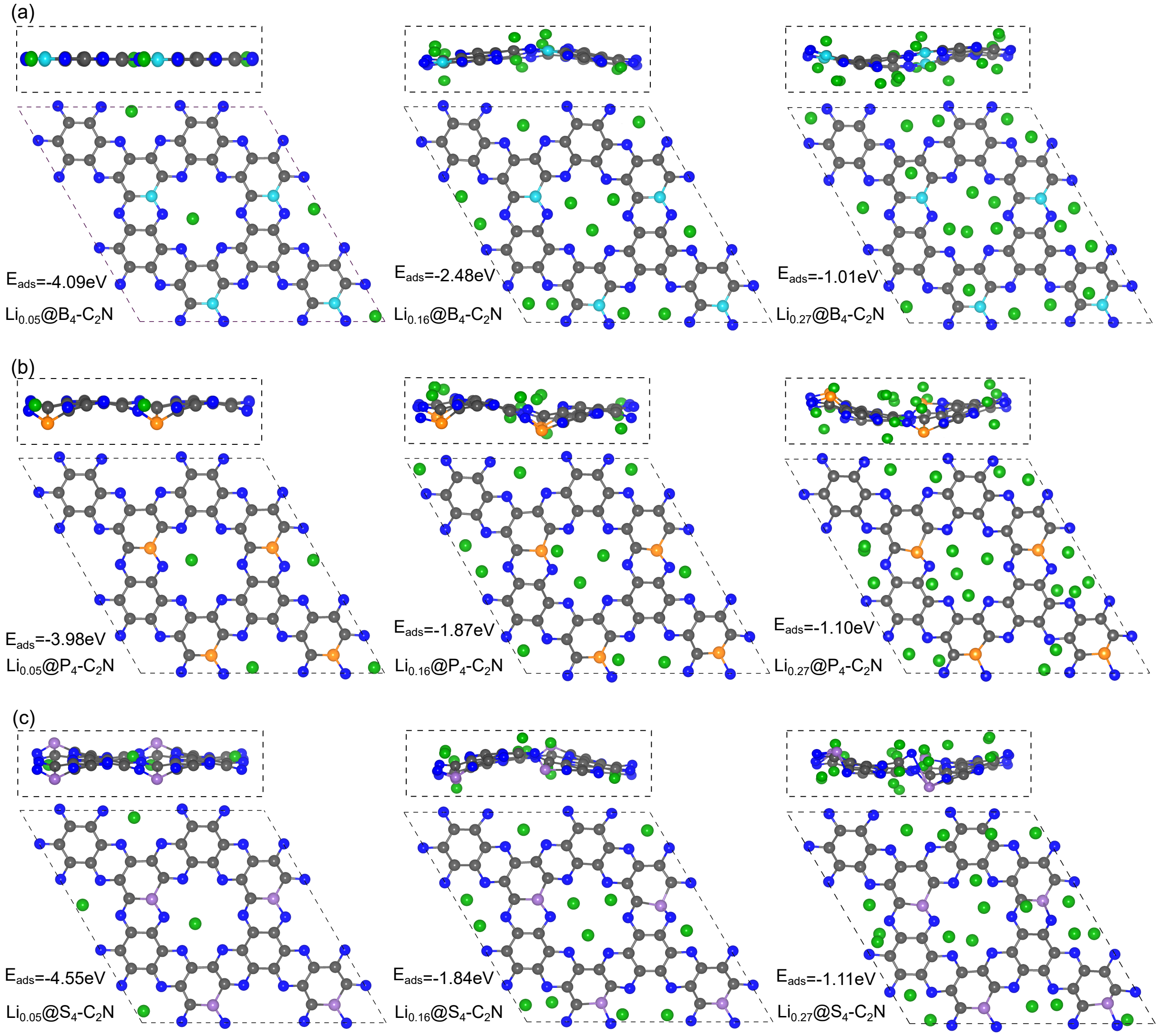

Figure S4: Top and side views of optimized Li adsorption structures on (a) B_4-C_2N, (b) P_4-C_2N, and (c) S_4-C_2N at varying concentrations. The corresponding adsorption energies (E_{ads}) are indicated for each configuration.

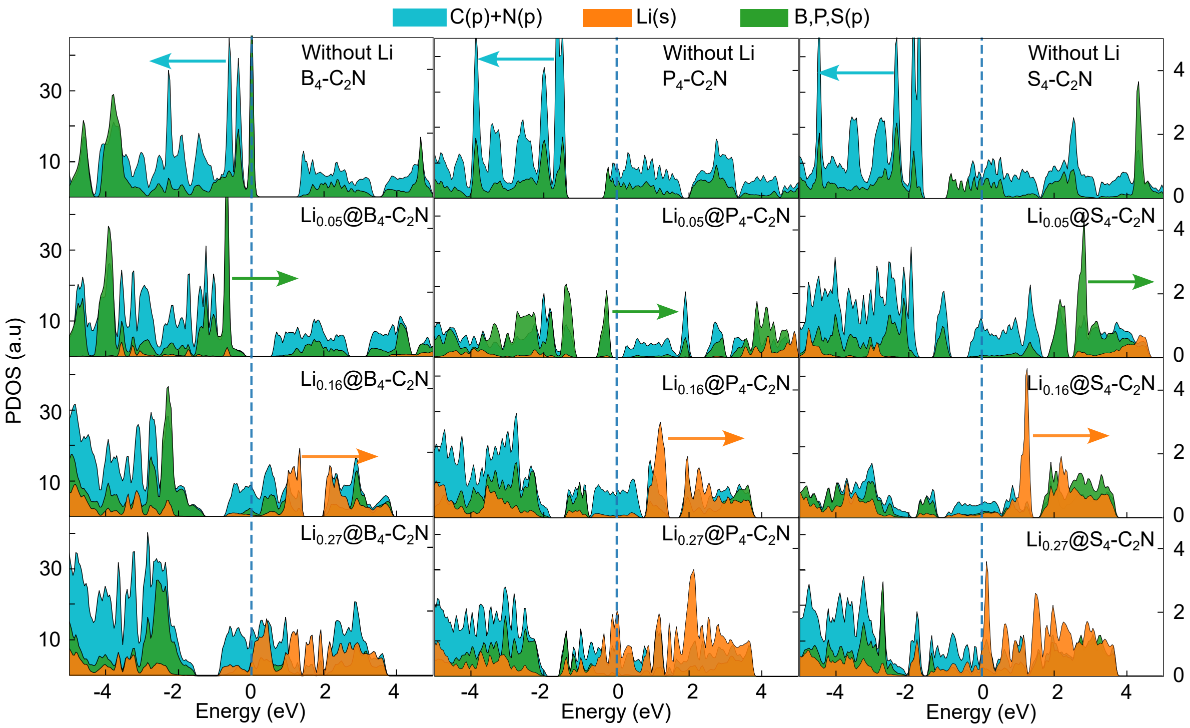

Figure S5: Projected density of states (PDOS) of X_4-C_2N (X = B, P, S) before and after lithiation at different concentrations. The turquoise, orange, and green shaded regions represent the contributions from C(p)+N(p), Li(s), and X(p), respectively. The turquoise arrow points to the left y-axis, which corresponds to the PDOS of C(p)+N(p), whereas the orange and green arrows refer to the right y-axis, representing the PDOS of Li(s) and X(p), respectively.

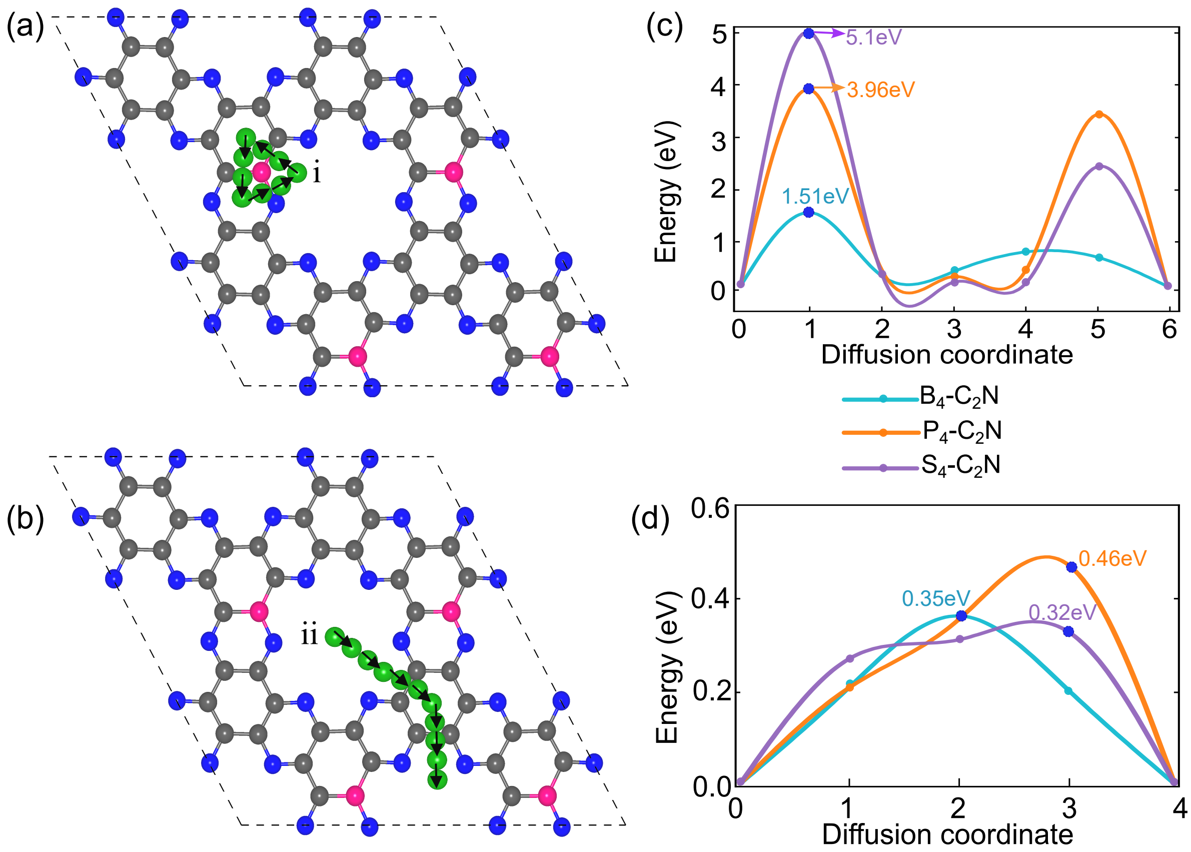

Figure S6: Li migration pathways and diffusion energy profiles in X_4–C_2N (X = B, P, S): (a,c) migration near dopant sites and (b,d) migration across doped pores via carbon-rich bridge rings. The blue markers denote the maximum diffusion barriers for X_4–C_2N (X = B, P, or S).

References

[1] Luo, J.-l.; Zhu, X.; Fan, L.; Chen, F.; Li, C.-m.; Li, G.-n.; Chen, Z.-Q. Transition metal (TM= Cr, Mn, Fe, Co, Ni) doped phosphorene as anode material for lithium-ion batteries predicted from first-principle calculations. *Computational Materials Science* **2020**, *183*, 109877.

[2] Wang, Y.; Xie, L.; Huang, R.; Yan, S.; Xie, X.; Zhang, Q. Theoretical investigation of Janus Ti_2BST (T= O, Se) monolayers as anode materials for Na/K-ion batteries. *Physical Chemistry Chemical Physics* **2024**, *26*, 18394–18401.

[3] Daula Shamim, S. U.; Hossain, M. K.; Hasan, S. M.; Hossain, A.; Ahmed, F. Ab initio study of N-doped graphene oxide (NDGO) as a promising anode material for Li-ion rechargeable battery. *Molecular Simulation* **2020**, *46*, 1135–1145.

[4] Dong, Y.; Wei, W.; Lv, X.; Huang, B.; Dai, Y. Semimetallic Si_3C as a high capacity anode material for advanced lithium ion batteries. *Applied Surface Science* **2019**, *479*, 519–524.

[5] Sannyal, A.; Ahn, Y.; Jang, J. First-principles study on the two-dimensional siligene (2D SiGe) as an anode material of an alkali metal ion battery. *Computational Materials Science* **2019**, *165*, 121–128.

[6] Wan, M.; Zhao, S.; Zhang, Z.; Zhou, N. Two-dimensional BeB_2 and MgB_2 as high capacity Dirac anodes for Li-ion batteries: A DFT study. *The Journal of Physical Chemistry C* **2022**, *126*, 9642–9651.

[7] Song, D.-X.; Xie, L.; Zhang, Y.-F.; Lu, Y.; An, M.; Ma, W.-G.; Zhang, X. Multilayer ion load and diffusion on TMD/MXene heterostructure anodes for alkali-ion batteries. *ACS Applied Energy Materials* **2020**, *3*, 7699–7709.

[8] Hu, Y.; Wang, J.; Lin, H. Metallic two-dimensional P_2C_3: a promising flexible anode for high-performance potassium-ion batteries. *Colloids and Surfaces A: Physicochemical and Engineering Aspects* **2021**, *619*, 126536.

[9] Makaremi, M.; Mortazavi, B.; Rabczuk, T.; Ozin, G. A.; Singh, C. V. Theoretical investigation: 2D N-graphdiyne nanosheets as promising anode materials for Li/Na rechargeable storage devices. *ACS Applied Nano Materials* **2018**, *2*, 127–135.
